# Discovering genetic determinants for cell-to-cell adhesion in two prevalent conjugative lactococcal plasmids

**DOI:** 10.1016/j.crmicr.2024.100239

**Published:** 2024-04-23

**Authors:** Guillermo Ortiz Charneco, Philip Kelleher, Andrius Buivydas, Paul P. de Waal, Irma M.H. van Rijswijck, Noël N.M.E. van Peij, Christian Cambillau, Jennifer Mahony, Douwe Van Sinderen

**Affiliations:** a School of Microbiology & APC Microbiome Ireland, University College Cork, Western Road, Cork, Ireland; b dsm-firmenich; Taste, Texture & Health, Center for Food Innovation, Alexander Fleminglaan 1, 2613 AX Delft, the Netherlands; c Laboratoire d'Ingénierie des Systèmes Macromoléculaires (LISM), Institut de Microbiologie, Bioénergies et Biotechnologie (IMM), Aix-Marseille Université – CNRS, UMR 7255, Marseille, France

**Keywords:** Lactococci, Conjugation, Adhesion, Gram-positive, Fluorescence, Cell clumping

## Abstract

Plasmids pNP40 and pUC11B encode two prevalent yet divergent conjugation systems, which have been characterized in detail recently. Here, we report the elucidation of the putative adhesins of the pNP40 and pUC11B conjugation systems, encoded by *traAd* and *trsAd*, respectively. Despite their significant sequence divergence, TraAd and TrsAd represent the most conserved component between the pNP40- and the pUC11B-encoded conjugation systems and share similar peptidoglycan-hydrolase domains. Protein structure prediction using AlphaFold2 highlighted the structural similarities between their predicted domains, as well as the potential homo-dimeric state of both proteins. Expression of the putative surface adhesins resulted in a cell clumping phenotype not only among cells expressing these surface adhesins but also between adhesin-expressing and non-producing cells. Furthermore, mutant derivatives of plasmids pNP40 or pUC11B carrying a mutation in *traAd* or *trsAd*, respectively, were shown to act as efficient donors provided the corresponding recipient expresses either *traAd* or *trsAd*, thus demonstrating *in trans* reciprocal complementarity of these proteins in conjugation systems.

## Highlights

• Two predicted peptidoglycan-hydrolases as potential adhesins in lactococcal conjugation.
• Overexpression of Tra11 and Trs15 cause a cell clumping phenotype in Lactococcus.
• Both proteins cross-complement each other, supporting interchangeable function.

## Graphical abstract

**Figure:**
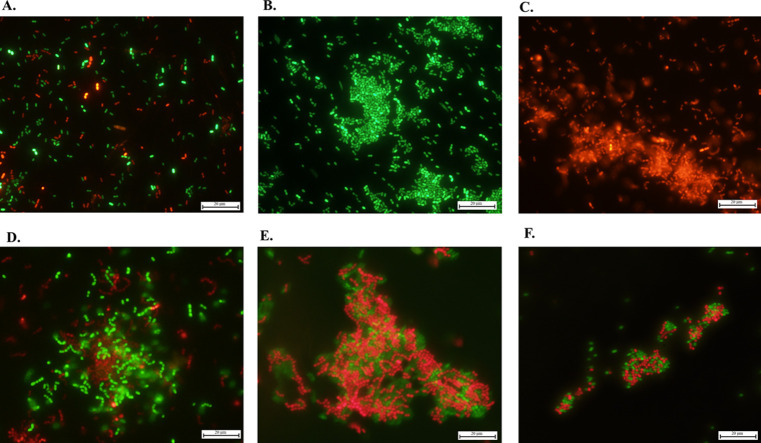

## 1 Introduction

Lactococci, and in particular strains belonging to the *Lactococcus lactis* and *Lactococcus cremoris* species, represent the most widely used starter cultures in the fermentation of dairy products. This is, in many respects, due to their characteristically abundant plasmid content which is known to encode relevant and technologically desirable traits (e.g. exopolysaccharide production, lactose metabolism, protease activity and bacteriophage resistance) (Mills et al., 2006; Fallico *et al*., 2012; Ainsworth *et al*., 2014). *L. lactis* strains typically carry multiple plasmids (from 4 to 12 different plasmids per strain), ranging in size from 0.87 kb to more than 100 kb (Cui *et al*., 2015; Van Mastrigt, *et al*., 2018; Kelleher *et al*., 2019), with significantly higher average plasmid content in dairy strains (*L. lactis* subsp. *cremoris, L. lactis* subsp. *lactis* and *L. lactis* subsp. *lactis* biovar *diacetylactis*) when compared to plant-derived strains (Kelly *et al*., 2010). Horizontal transfer of plasmids among *L. lactis*/*cremoris* strains is currently achieved predominantly by conjugation and transduction (Ainsworth *et al*., 2014). Conjugation among lactococcal strains represents a very useful, non-recombinant tool for the development of novel strains with desirable traits suited to the dairy environment (Ortiz Charneco *et al*., 2023a).

Despite the economic significance of these Gram-positive lactococcal species, the molecular process of conjugation has been more extensively studied in Gram-negative organisms when compared to their Gram-positive counterparts. In Gram-negative bacteria, conjugation relies on pilus formation to establish cell-to-cell contact between donor and recipient, whereas Gram-positive bacteria appear to rely on cell surface-associated adhesins (Kohler *et al*., 2019). For example, the conjugative enterococcal plasmid pCF10 encodes three surface proteins, PrgA, PrgB and PrgC, with PrgA providing surface exclusion and thus preventing plasmid transfer between donor strains (Schmitt *et al*., 2020), and PgrB being responsible for promoting a distinct cellular aggregation phenotype (Bhatty *et al*., 2015). Similarly, in the model Gram-positive conjugative plasmid pIP501 from *Enterococcus faecalis*, TraO has been proposed to act as the surface adhesin (Goessweiner-Mohr *et al*., 2014; Kohler *et al*., 2018), and more recently TIE_pLS20_ has been identified as the surface adhesin from the *Bacillus subtilis* conjugative plasmid pLS20 (Gago-Córdoba et al., 2021). The precise mechanism of action that facilitates adhesion between donor and recipient cells of these two previously identified proteins remains still unknown. No surface adhesin has as yet been identified for lactococcal conjugation systems, with perhaps one exception: the cell-membrane-anchored protein CluA, which is encoded by the integrative conjugative element (ICE) or sex factor of *L. cremoris* MG1363, and which is required for high frequency conjugation, promotes cell-to-cell contact and causes a cell clumping phenotype (Wels *et al*., 2019).

A recent study revealed the presence of 33 lactococcal plasmids among public databases with conjugation-related genes similar to those of previously described lactococcal conjugative plasmids pMRC01, pAF22 and pNP40 (Coakley *et al*., 1997; O'Driscoll *et al*., 2004; Fallico *et al*., 2012; Ortiz Charneco *et al*., 2021). The conjugation-associated genes of plasmids pUC11B and pNP40 have recently been characterized, although the functionality responsible for cell-to-cell contact was not unambiguously identified (Ortiz Charneco *et al*., 2021, 2023b). The traAd and trsAd genes have been suggested to encode the surface adhesin of the pNP40- and pUC11B-associated conjugation machinery, respectively, based on the presence of conserved N-acetylmuramoyl-l-alanine amidase domains. These domains have been reported to be a characteristic of proteins with bifunctional roles as both cell wall hydrolases and adhesins, such as Rv3717 from *Mycobacterium tuberculosis* (Kumar *et al*., 2013) and other Gram-positive pathogenic bacteria, such as Aas from *Staphylococcus saprophyticus* (Henderson and Martin, 2011) and Aaa from *Staphylococcus aureus* (Hirschhaysen *et al*., 2012). Among these previously described proteins, only Aaa possessess a LysM domain for binding to peptidoglycan, whereas neither Rv3717 nor Aas possess a recognizable cell wall binding domain despite their adhesion properties.

Mutation of the genes encoding TraAd and TrsAd causes a major reduction in the conjugation frequency of their respective conjugative plasmids (up to 1000- and 4500-fold, respectively) (Ortiz Charneco *et al*., 2021, 2023b) suggesting an important role in conjugation. In the current report we performed an experimental and predictive analysis of the role played by TraAd and TrsAd in conjugation. The generated results implicate TraAd and TrsAd as adhesins involved in cell-to-cell contact during conjugation.

## 2 Materials and methods

### 2.1 Bacterial strains and growth conditions

Bacterial strains employed in this study are summarised in Supplementary Table S1. Overnight cultures were incubated at 30 °C for 16 h by inoculating bacterial cells from -80 °C glycerol stocks into 10 mL of M17 supplemented with 0.5 % (v/v) glucose (GM17) containing either nisin (2.5 μg/mL, for selection of pNP40), tetracycline (10 μg/mL, to select strains harbouring pUC11B or pPTPi) or erythromycin (5 μg/mL, which selects for strains containing pPEPi, pGFP8048E or pMC8048E). Electrocompetent cells of *L. cremoris* were prepared as previously described (Holo and Nes, 1989).

### 2.2 Plasmids and constructs

All plasmids employed in this study are summarised in Supplementary Table S2. Purification of plasmid DNA was performed using the GeneJET Plasmid MiniPrep Kit and following the manufacturer's instructions (Thermo Scientific, USA). Newly constructed plasmids were generated by conventional recombinant DNA techniques. PCR fragments used for cloning (Supplementary Table S3) were digested with the same enzymes as the relevant cloning vector, and then ligated with T4 DNA ligase. Ligation mixtures were subsequently introduced into competent *L. cremoris* NZ9000 (Kuipers *et al*., 1998) by electroporation and transformants were screened by colony PCR to confirm the presence of both plasmid and insert. Finally, the integrity of the construct sequence was verified by Sanger sequencing (Genewiz, Azenta Life Sciences, Leipzig, Germany).

The fluorescent protein-based reporter plasmids pGFP8048E and pMC8048E were created using the backbone of plasmid pNZ8048E, which was in turn constructed using the backbone of pNZ8048 (De Ruyter et al., 1996) and the erythromycin resistance gene from pNZ44E (Draper *et al*., 2009; Ortiz Charneco *et al*., 2024). The GFP-encoding gene from the fusion vector pHTP9 and the mCherry-encoding gene from the fusion vector pHTP1-mCherry (NZYTech, Portugal) were individually PCR-amplified allowing the incorporation of an artificial Shine-Dalgarno sequence at the 5′-end and a stop codon at the 3′-end of these genes, using oligonucleotides listed in Supplementary Table S3. These PCR-generated DNA fragments were subsequently digested with the same enzymes as vector pNZ8048E and ligated using T4 DNA ligase (Promega). Induced expression of these genes was achieved by the inclusion of nisin (10 ng/ml) in the growth medium. The proposed surface adhesin-encoding genes, *traAd* and *trsAd*, were PCR-amplified (incorporating an artificial Shine-Dalgarno sequence at the 5′-end of the gene) and individually cloned into the low-copy expression vectors pPTPi (O'Driscoll *et al*., 2004) and pPEPi (Ortiz Charneco *et al*., 2023b), generating constructs pPTPi::*traAd*, pPTPi::*trsAd*, pPEPi::*traAd* and pPEPi::*trsAd*. These genes were cloned so that their transcription is under the control of the nisin-inducible promoter P*nisA* being present in both pPTPi and pPEPi, and thus transcription of the relevant gene was induced by adding 10 ng/ml of nisin to the growth medium at the point of inoculation.

### 2.3 Mating assays

Conjugation was performed using the spread solid mating approach, as previously described (Ortiz Charneco *et al*., 2021). Briefly, overnight cultures of both donor and recipient were mixed in a 1:1 volume ratio (representing a 1:1.5 donor/recipient viable cell count ratio), centrifuged at 3000× *g*, resuspended in 200 µL of 5 % reconstituted skim milk (RSM) supplemented with 2 % glucose and evenly spread on 5 % RSM, 2 % glucose agar plates. Plates were subsequently incubated overnight at 30 °C, after which the cells were scraped from the plates and suspended in 4 mL of Ringer's solution, serially diluted and plated onto GM17 agar plates supplemented with the relevant antibiotic.

To determine the possible role of TraAd and TrsAd as surface adhesins of the pNP40 and pUC11B conjugation systems, respectively, constructs pPEPi::*traAd* and pPEPi::*trsAd* were individually introduced into *L. cremoris* NZ9000, and the resulting strains were subsequently used as recipients in spread solid mating conjugation experiments using various donors. The donor strains applied were *L. cremoris* NZ9000 strains carrying pNP40 or pUC11B, or *traAd^−^* or *trsAd^−^* derivatives of these respective plasmids (mutant nomenclature *L. cremoris* NZ9000 *traAd*::Ter or *L. cremoris* NZ9000 *trsAd*::Ter, respectively). The mutant derivatives of pNP40 and pUC11B had been constructed previously using an ssDNA recombineering approach (van Pijkeren and Britton, 2012; Ortiz Charneco *et al*., 2021, 2023b). Overall, these mutants were constructed so as to incorporate two consecutive stop codons within the first 100 bps of either *traAd* in pNP40 or *trsAd* in pUC11B, thereby disrupting their expression. Prior to mixing donor and recipient cells, expression of the proposed surface adhesins was induced in the recipient cultures by adding 10 ng/ml nisin to the growth medium at the point of inoculation.

### 2.4 Cell clumping assay

To ascertain if expression of the presumed surface adhesin-encoding genes, *traAd* and *trsAd*, promoted a cell clumping phenotype in bacterial cells expressing these proteins, two fluorescent reporter plasmids were constructed, pGFP8048E and pMC8048E, producing green and red fluorescent proteins, respectively. These were introduced individually into *L. cremoris* NZ9000 pPTPi (control), *L. cremoris* NZ9000 pPTPi::*traAd* and *L. cremoris* NZ9000 pPTPi::*trsAd*. The resulting strains were used in different combinations in subsequent cell clumping assays. For cell clumping assays, overnight cultures were diluted (on their own or in different strain combinations) 1:100 in 10 ml fresh GM17 supplemented with the relevant antibiotics and 10 ng/ml of nisin for induction of the surface adhesins and/or the fluorescent proteins and grown to an OD_600nm_ of 1.5. A variety of induction conditions were evaluated including time of addition (from 0 to 120 min) and the nisin concentration (1–20 ng/ml), and those stated above represent the optimal conditions identified for the restoration of the *traAd* and *trsAd* mutants and the observed cell clumping phenotype. Subsequently, 10 µL of each culture was spotted on the middle of a microscope slide, covered with a cover slip following the wet mount method and visualized using a 60X objective lens of an upright fluorescence microscope (BX53; Olympus) for imaging.

### 2.5 Comparative and functional analysis

Sequence comparison at the protein level was performed using bi-directional BLAST alignment (Altschul *et al*., 1990). TMHMM v2.0 software was employed for the prediction of transmembrane helices using the hidden Markov model (HMM) (Krogh *et al*., 2001). HHpred software was used for remote protein homology detection and structure prediction with pairwise comparison of profile HMM (Söding *et al*., 2005; Zimmermann *et al*., 2018). Pfam (El-Gebali *et al*., 2019) was used for the identification of functional domains. Jalview V. 2.11 Desktop was employed to perform Clustal Omega alignment of the TraAd and TrsAd proteins (Procter *et al*., 2021), while SignalP 6.0 was utilised for the prediction of secretion signals in protein sequences (Teufel *et al*., 2022).

### 2.6 Protein structure predictions

We performed predictions using either a Colab notebook running AlphaFold (Jumper *et al*., 2021) (https://colab.research.google.com/github/deepmind/alphafold/blob/main/notebooks/AlphaFold.ipynb) on Nvidia A100 40 Gb GPU. Local Distance Difference Test (LDDT) evaluates local distance differences of all atoms in a model with reference to an ensemble of equivalent structures. The pLDDT (predicted LDDT-Cα) is a per-residue measure of local confidence on a scale from 0 to 100 (100 being the highest confidence level). The PAEs plots were used to assess the quality of the interactions within our assemblies. The pLDDT values that are stored in the pdb file as B-factors, were plotted using Excel. Visual representations of the structures were prepared with ChimeraX (Pettersen *et al*., 2021).

### 2.7 Statistical data analysis

Data in this study represent the means ± standard deviation (SD) of triplicate assays. Results were analysed using the SigmaPlot 11.0 statistical package (SPSS), from Systat Software, Inc., San Jose California USA. One-way analysis of variance ANOVA was performed to compare frequencies of conjugation. A *P* value of ≤0.001 was considered very significant and is represented by two asterisks “**” in the graphs.

### 2.8 Data availability statement

Coordinates of predicted structures are accessible on Zenodo.

## 3 Results

### 3.1 Comparative genomics and domain comparison

The mechanism of plasmid conjugation in lactococcal species has been the subject of two recent studies (Ortiz Charneco et al., 2021, 2023b). However, a critical component of lactococcal plasmid-mediated conjugation remains elusive: the surface adhesins responsible for promoting the essential initial contact between donor and recipient cells during conjugation. Thus, the objective of this study was to address this gap in our knowledge and enhance our understanding of the adhesion process during plasmid-mediated conjugation in lactococcal species. Plasmids pNP40 and pUC11B have recently been determined to represent two of the most prevalent lactococcal conjugative systems among public databases (Ortiz Charneco *et al*., 2021). The pNP40 and pUC11B conjugation systems were selected for further characterization in this study based on their sequence divergence, thus representing potentially distinct systems, and prevalence among public databases.

Previous functional analyses have elucidated several analogous functions between the two conjugation systems, despite their overall low sequence similarity (Ortiz Charneco *et al*., 2023b). Despite the overall divergence, both conjugation gene clusters share a set of three adjacent genes whose products exhibit relatively high amino acid identity (Supplementary Figure S1). The first of this triplet, i.e. *traAd* and *trsAd* for pNP40 and pUC11B, respectively, encode predicted cell wall-degrading proteins, with 67.68 % amino acid identity. The second, represented by *tra10_pNP40_* and *trs16_pUC11B_* (located on pNP40 and pUC11B, respectively), encode predicted mating channel-related proteins sharing 31.25 % amino acid identity, while the third, i.e. *tra09* in pNP40 and *trs17* in pUC11B, encode predicted thioredoxin-like proteins sharing 54.46 % amino acid identity. Apart from these genes, the pNP40 and pUC11B conjugation gene clusters share little protein sequence similarity.

Despite recent functional studies, no cell surface adhesin-encoding genes have yet been proposed for either the pNP40- or the pUC11B-associated conjugation systems. Based on the presence of N-acetylmuramoyl-l-alanine amidase domains in TraAd and TrsAd (described in the next paragraph), and their similarity to amidase domains present in a small fraction of surface adhesins of chromosomally encoded ICEs from Gram-positive bacteria (Hirschhausen *et al*., 2012; Kumar *et al*., 2013; Wels *et al*., 2019), these two proteins were targeted for subsequent experiments as potential surface adhesins of their respective conjugation systems. Mutation of the corresponding genes had been shown to significantly diminish conjugation frequencies of pNP40 and pUC11B (Ortiz Charneco *et al*., 2021, 2023b).

**Fig. 1:**
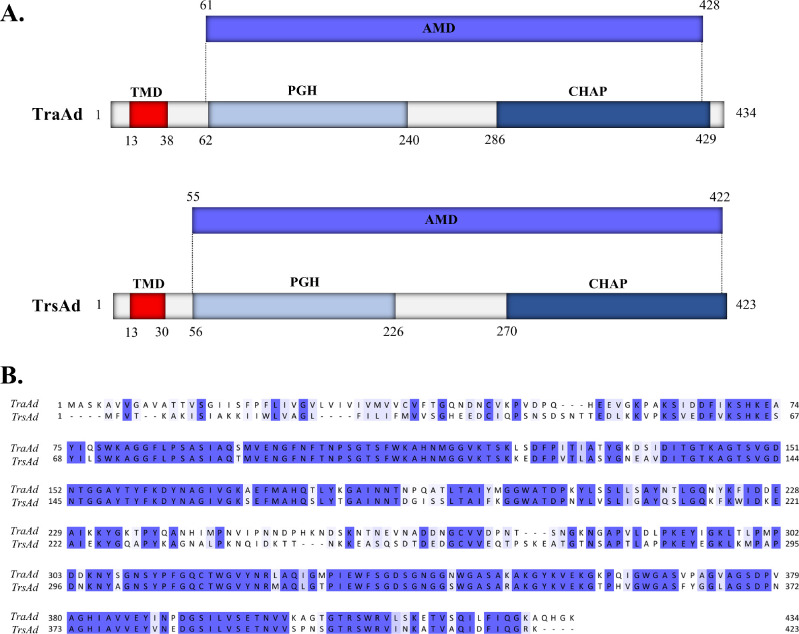
**(A)** Functional domains present in TraAd and TrsAd, based on sequence and structural similarities using HHpred, TMHMM and Pfam databases; **TMD**: transmembrane domain. **PGH**: peptidoglycan hydrolase domain. **CHAP**: cysteine, histidine-dependent amidohydrolase/peptidase domain. **AMD**: N-acetylmuramoyl-l-alanine amidase domain. **(B)** Jalview of a Clustal Omega alignment for the TraAd and TrsAd proteins. Conserved amino acids are highlighted with two shades of blue based on conservation, (light blue aminoacid with strongly similar properties; dark blue: aminoacid fully conserved) while uncoloured amino acids correspond to non-conserved regions.

A predictive analysis was performed using HHpred (Zimmermann *et al*., 2018), TMHMM (Krogh *et al*., 2001) and Pfam (El-Gebali *et al*., 2019) in TraAd (434 aa) and TrsAd (423 aa) to search for protein similarity among public databases, the results of which are summarized in Fig. 1A. Based on TMHMM analyses (Krogh *et al*., 2001), both proteins contain a transmembrane domain in their N-terminal end, which may act as a membrane anchor, followed by an N-acetylmuramoyl-l-alanine amidase-like domain encompassing much of the remainder of the protein sequence. This latter domain is further divided in two sub-domains, i.e. a peptidoglycan-glycosyl hydrolase (PGH) domain (closer to the N-terminal end), followed by a linker of 44 amino acids, and a cysteine, histidine-dependent amidohydrolase/peptidase (CHAP) domain in their C-terminal end. Alignment of the TraAd and TrsAd sequences using the Clustal Omega package (Fig. 1B) revealed that these two sub-domains (i.e., PGH and CHAP domain) are conserved between the two proteins. Neither TraAd nor TrsAd presented any significant signal peptidase signature following their transmembrane domains.

**Fig. 2:**
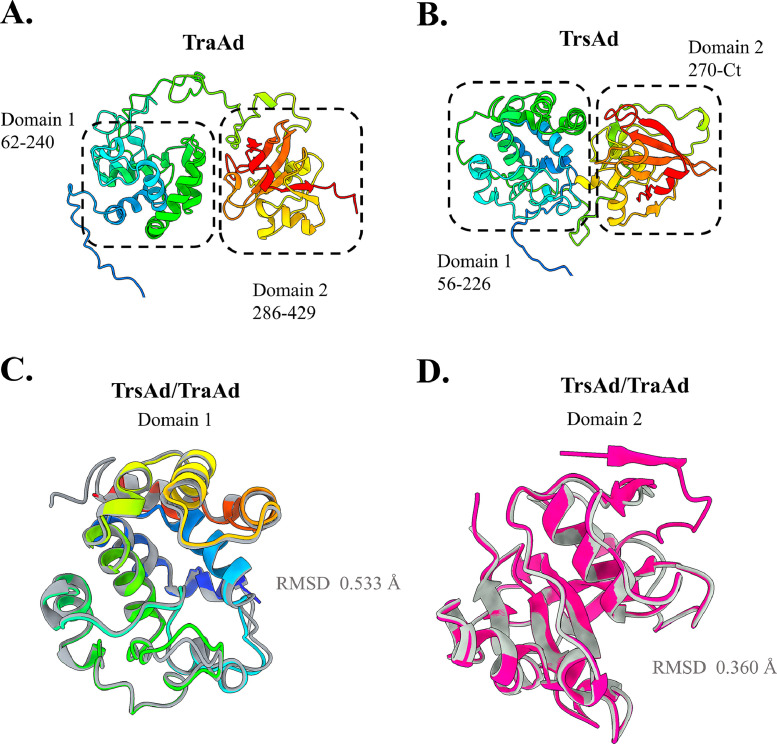
TraAd and TrsAd predicted structures and domain comparison. **(A)** Ribbon representation of the predicted structure of TraAd, rainbow-coloured from N-terminus to C-terminus. The two predicted domains are outlined in dotted rectangles. **(B)** Ribbon representation of the predicted structure of TrsAd, rainbow-coloured from N-terminus to C-terminus. The two predicted domains are indicated by dotted rectangles. **(C)** Superposition of the Domain 1 from TrsAd (coloured) and TraAd (grey). **(D)** Superposition of the Domain 2 from TrsAd (coloured) and TraAd (grey). The TMHMM predicted and disordered trans-membrane domain in the N-terminus of TraAd and TrsAd were removed. Abbreviations: **Ct**: C-terminus; **RMSD:** Root Mean Square Deviation.

A structure prediction of TraAd (434 aa) and TrsAd (423 aa) was implemented using AlphaFold2 (Jumper *et al*., 2021) to further assess functional domains (Fig. 2A & B). The Dali server reported a convincing match between the TraAd and TrsAd peptidoglycan-hydrolase (domain 1, Fig. 2C) and CHAP domains (domain 2, Fig. 2D) (root mean square deviation (RMSD) of 0.533 Å between their domain 1 and 0.360 Å between their domain 2), denoting their quasi-identical structures.

**Fig. 3:**
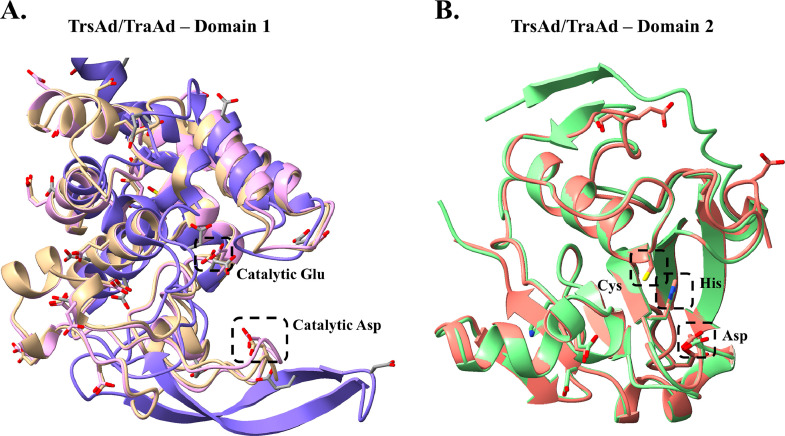
TraAd and TrsAd predicted catalytic domains. **(A)** Superposition of the Domain 1 from TrsAd (pink) and TraAd (beige) with the autolysin domain of L. monocytogenes (PDB: 3FI7). The catalytic residues Glu/Asp are marked by dotted rectangles. **(B)** Superposition of the Domain 2 from TrsAd (green) and TraAd (orange). The catalytic residues Cys/His/Asp are marked by dotted rectangles.

Domain 1 and domain 2 possess putative catalytic residues, a Glu/Asp (residues 96/151 and 89/144 for TraAd and TrsAd, respectively) catalytic dyad for domain 1 (Fig. 3A), and a Cys/His/Asp (residues 317/382/377 and 310/375/370 for TraAd and TrsAd, respectively) catalytic triad for the second domain (Fig. 3B). Domain 1 was superimposed onto a known structure, i.e. the autolysin of *Listeria monocytogenes* (PDB: 3FI7), to identify the catalytic acidic amino acids. The *Listeria* autolysin presents two catalytic residues Glu/Glu (residues 122/156) (Bublitz *et al*., 2009). Domains 1 and 2 of TraAd and TrsAd are both expected to participate in degradation of the cell-wall peptidoglycan by hydrolyzing the polysaccharide bonds and the amido bond, respectively.

**Fig. 4:**
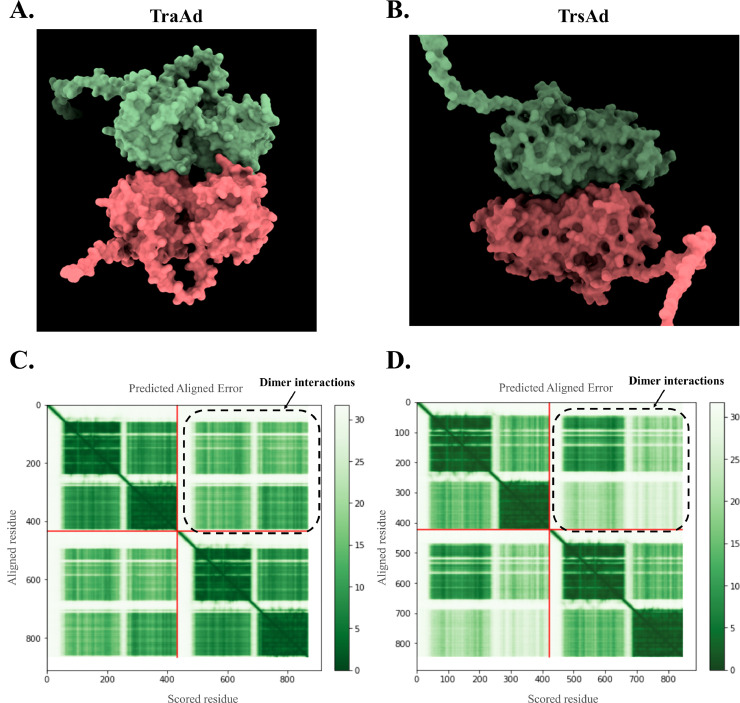
TraAd and TrsAd predicted dimerisation. **(A)** Spatial structure of the TraAd homodimer. **(B)** Spatial structure of the TrsAd homodimer. **(C, D)** Heatmap of the predicted aligned error (PAE) of TraAd and TrsAd, respectively. Colour at the “x” and “y” axis indicates AlphaFold2’s expected position error at residue “x” if the predicted structures were aligned on residue “y”.

TraAd (Fig. 4A) and TrsAd (Fig. 4B) were predicted by AlphaFold2 to form a complex of identical dimers (homodimers). Predicted aligned error (PAE) plots of TraAd (Fig. 4C) and TrsAd (Fig. 4D) suggest that each monomer conformation is not only predicted with confidence, but that homodimer packing is of relatively high confidence too. It is noteworthy that the performance of AlphaFold2 is better for homo-oligomer prediction than for hetero-oligomer prediction, since for the former the contact surface is usually larger compared to what would generally be the case for the latter (Xiong et al., 2022). The pLDDT values were stored as B-factors (Supplementary Figure S2).

### 3.2 Two potential surface adhesins restore a conjugation deficiency phenotype of corresponding mutants through reciprocal, *in trans* complementation

**Fig. 5:**
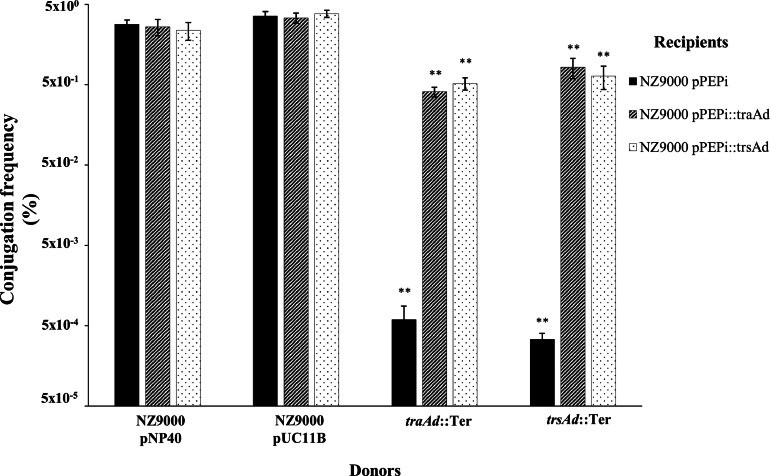
Conjugation frequencies between *L. cremoris* NZ9000 pNP40, pUC11B and the adhesin-deficient derivatives as donors strains against the recipients *L. cremoris* NZ9000 pPEPi, pPEPi::*traAd* or pPEPi::*trsAd*. Frequency of conjugation of the mutated version of either pNP40 or pUC11B is compared against the frequency of conjugation of the respective wild-type plasmid. A *P*-value ≤ 0.001 was considered very significant and is represented by two asterisks “∗∗”. Presented data are the mean of three replicates ± standard deviation.

Following the *in silico* analysis, *traAd* and *trsAd* were selected as candidate surface adhesin-encoding genes. To experimentally evaluate the proposed surface adhesin functionality, *traAd* and *trsAd* were individually cloned into a nisin-inducible expression vector and introduced into *L. cremoris* NZ9000 and employed as recipients in conjugation experiments using a donor strain harbouring either the *traAd^−^* or *trsAd^−^* mutated derivatives of pNP40 or pUC11B, respectively. It was hypothesized that the adhesin could be expressed in either the donor or recipient cell since either would promote cell-to-cell contact. Therefore, a recipient cell expressing one of the proposed adhesins would be expected to restore (reciprocally and *in trans)* conjugation frequencies in an otherwise deficient conjugation process caused by a mutation of the proposed surface adhesin-encoding gene in the conjugative plasmid of the donor. Expression of *traAd* or *trsAd* (Materials and Methods; by adding 10 ng/mL of nisin to the growth medium) in a recipient *L. cremoris* NZ9000 resulted in very significant (*P* ≤ 0.001) increases in the conjugation frequencies between the negative control harbouring pPEPi and those harbouring the pPEPi::*traAd* and pPEPi::*trsAd* constructs (Fig. 5).

Conjugation frequencies of *traAd*::Ter increased from 5.97 × 10^−4^ ± 2.82 × 10^−4^ % (*L. cremoris* NZ9000 pPEPi recipient) to 4.1 × 10^−1^ ± 5.73 × 10^−2^ % with *L. cremoris* NZ9000 pPEPi::*traAd_pNP40_* as recipient, and to 5.2 × 10^−1^ ± 1.88 × 10^−1^ % with *L. cremoris* NZ9000 pPEPi::*trsAd_pUC11B_* as recipient, representing a 680-fold and 870-fold increase in conjugation frequency, respectively. Similarly, employing *trsAd*::Ter as donor, very significant (*P* ≤ 0.001) differences were observed in the conjugation frequencies between the recipient arbouring the empty vector (3.38 × 10^−4^ ± 6.44 × 10^−5^ %) and those expressing *trsAd* (6.47 × 10^−1^ ± 2.08 × 10^−1^ %) and *traAd* (8.3 × 10^−1^ ± 9.6 × 10^−2^ %), in which conjugation of plasmid pUC11B increased more than 1900-fold and 2400-fold, respectively.

Overall, conjugation frequencies of strains carrying *traAd* and *trsAd* mutations in the pNP40 and pUC11B conjugation gene cluster, respectively, increased very significantly (*P* ≤ 0.001) when intact copies of either gene were expressed *in trans* in a recipient strain. Furthermore, both genes displayed a similar reciprocal restorative effect in either mutant derivative of the two conjugation systems, indicative of an analogous function. Additionally, expression of either gene in a recipient cell during conjugation with a donor harbouring intact copies of either conjugative plasmid showed no significant (*P* ≤ 0.001) differences with the control.

### 3.3 The proposed surface adhesins promote a clumping phenotype

To further substantiate the hypothesis that TraAd and TrsAd represent surface adhesins, the genes encoding these proteins were individually cloned into the low copy vector pPTPi and introduced into *L. cremoris* NZ9000 strains harbouring one of the reporter plasmids, pGFP8048E, expressing the reporter Green Fluorescent Protein (GFP) or pMC8048E, expressing the red fluorescent reporter protein mCherry. These strains were subsequently co-cultured in different combinations to determine if any cell-to-cell contact could be observed after TraAd and/or TrsAd induction, which may or may not be visible in cultures, but which would be visible in microscopic images.

**Fig. 6:**
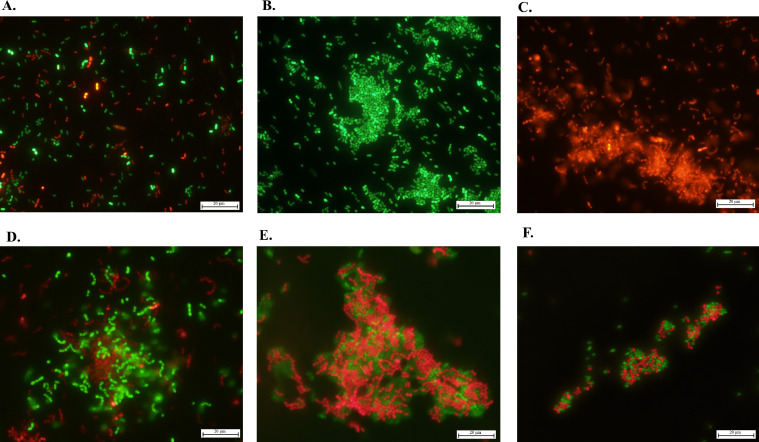
Fluorescent microscopy images of *L. cremoris* NZ9000 derivatives. **(A)** NZ9000 pPTPi (green) and NZ9000 pPTPi (red); **(B)** NZ9000 pPTPi::*traAd* (green); **(C)** NZ9000 pPTPi::*trsAd* (red); **(D)** NZ9000 pPTPi::*traAd* (green) and NZ9000 pPTPi (red); **(E)** NZ9000 pPTPi::*traAd* (green) and NZ9000 pPTPi:*trsAd* (red); **(F)** NZ9000 pPTPi (green) and NZ9000 pPTPi::*trsAd* (red).

When two strains were co-cultured, in which one strain harboured one of the reporter plasmids and the other strain harboured the empty vector pPTPi (Fig. 6A), no cell clumping was observed irrespective of nisin addition. However, *L. cremoris* NZ9000 cells expressing either *traAd* (Fig. 6B) or *trsAd* (Fig. 6C) showed a distinct clumping phenotype when cultured separately and after nisin induction. This was observed on imaging analysis, but it is worth noticing that this cell clumping phenotype could also be partially observed in the tubes, in which apparently broken-off and free-floating cell clumps could be seen after vortexing of the tubes (data not shown). Additionally, *L. cremoris* NZ9000 cells expressing *traAd* displayed cell clumping when co-cultured with *L. cremoris* NZ9000 cells not expressing either of the proposed surface adhesins (Fig. 6D), and similarly *L. cremoris* NZ9000 cells expressing TrsAd clumped together with *L. cremoris* NZ9000 cells (Fig. 6E). Finally, this clumping phenotype was also observed when *L. cremoris* NZ9000 cells producing TraAd were co-cultured together with *L. cremoris* NZ9000 pPTPi::*trsAd* (Fig. 6F). Of note, no cell clumping phenotype was observed between strains containing either the wild type pNP40 or pUC11B or their TraAd/TrsAd mutant derivatives and recipient cells of *L. cremoris* MG1614 (data not shown). This could be due to the strict regulation that both conjugation clusters are subjected to, and that expression levels of TraAd and TrsAd are significantly lower in a wild-type background when compared to their ectopic, nisin-induced overexpression.

## 4 Discussion

Despite the economic significance of lactococci for the dairy industry, lactococcal plasmid-encoded conjugation has been less extensively characterized compared to certain other Gram-positive conjugative plasmids, such as the enterococcal model plasmids pCF10 and pIP501 **(**Goessweiner-Mohr et al., 2014**;** Kohler et al., 2019). The lactococcal conjugative plasmids pNP40 and pUC11B have been subject to recent characterization studies **(**Ortiz Charneco et al., 2021, 2023b), which showed that both plasmids contain two prevalent yet distinct gene clusters responsible for conjugation. Unraveling their mechanistic details will provide insights into other similar systems present among conjugative plasmids present in lactococci and other Gram-positive bacteria.

No conserved domains were observed between TraAd or TrsAd and the characterized chromosomally encoded clumping factor in *L. cremoris* MG1363, CluA (Stentz *et al*., 2006), or to other surface adhesins such as PrgC in the *E. faecalis* pCF10 plasmid (Bhatty *et al*., 2015) and TraO, the surface adhesin of the model Gram-positive conjugative plasmid, pIP501 (Kohler *et al*., 2018). For instance, TraO, PrgC and CluA were shown to possess transmembrane domains in their C-terminal ends, highly repetitive sequence motifs of three-residue periodicity and an LPxTG cell wall anchor motif (Navarre and Schneewind, 1999; Goessweiner-Mohr *et al*., 2014). Neither TraAd nor TrsAd presented any significant similarities to these proteins. Beyond their transmembrane domain located in their amino-terminus, no mechanism of action that would promote cell-to-cell binding has been observed. It is noteworthy that the cell wall in lactic acid bacteria has only been studied in recent years, and that characterization efforts regarding surface adhesins in lactococcal species have been scarce (Chapot-Chartier, 2014).

Our results provide evidence to the possible role of proteins TraAd and TrsAd as surface adhesins, and their function appears to be interchangeable between the two conjugation systems which are otherwise divergent on a protein similarity level from each other beyond the previously discussed gene triplet (Supplementary Figure S1).. It was observed that cells expressing either TraAd or TrsAd display a cell clumping phenotype against cells not expressing either of these proteins. Additionally, cells expressing TraAd were observed to clump together with cells expressing TrsAd, and *vice versa*. These results suggest that either there is a different exclusion mechanism preventing mating of two donor cells during conjugation that is not related to the presence of these proposed surface adhesins in two different cells, or that there is no exclusion mechanism in lactococcal conjugation. Ectopic overexpression of either of the two proteins did not cause any visible cell lysis effects, neither on cell growth nor under the microscope, which could have been otherwise expected from two proteins with amidase domains. We hypothesize that if such lysis is taking place within the cells, this may be localized and apparently not lethal to the cell overexpressing these proteins.

The amidase/hydrolase modules present in TraAd and TrsAd may be related to a VirB1 role. VirB1 acts as a periplasmic transglycosylase, creating breaks within the cell wall of *A. tumefaciens* to facilitate the assembly of its Type IV Secretion System (T4SS) (Cook and Herman, 2023). This notion is supported by the lack of any other pNP40- or pUC11B-encoded conjugation-related protein that could function as VirB1 (Supplementary Figure S1), which suggests that TraAd and TrsAd are responsible for “punching holes” in the peptidoglycan wall to facilitate the assembly of the conjugation machinery. This peptidoglycan degradation role may be an indication that TraAd and TrsAd are moonlighting proteins, since they would both act as hydrolases and adhesins, or that the predicted amidase activity is alone responsible for adhesion between cells in a yet uncharacterized manner. Previously described adhesins, PgrB from plasmid pCF10 (Bhatty *et al*., 2015) and TraO from pIP501(Kohler *et al*., 2018), present an N-terminal signal-peptide domain and a C-terminal LPxTG cell wall anchor motif, responsible for recognizing and binding to the cell wall of Gram-positive bacteria. Similarly, VirB1 possesses a signal-peptide domain in its N-terminal, followed by a lytic transglycosylase domain, responsible for creating disruptions in the peptidoglycan strands necessary for the assembly of the *A. tumefaciens* conjugation machinery, though it does not contain an LPTxTG domain. None of these domains are present in neither TraAd nor TrsAd, which could further substantiate their similarity to VirB1.

TraAd and TrsAd do not contain known cell wall-binding domains, which makes it hard to determine their mechanism of action. Based on their predicted three-dimensional structure, their catalytic domains and the observed conjugation and clumping assays, they may weaken the cell wall on the donor strain to facilitate anchoring of the conjugation machinery as mentioned above. This presumption is consistent with our (reciprocal) *in trans* complementation of conjugation of adhesin-deficient versions, although conjugation frequencies are not completely restored, which may be due to the inherent obstacles of *in trans* complementation, imperfect timing or suboptimal level of expression. After weakening the donor's peptidoglycan layer, they could protrude from the cell wall, exposing their peptidoglycan hydrolase and CHAP domains to the extracellular environment. Upon contact with other possible recipient cells, these domains may be responsible for the degradation of the peptidoglycan layer of these recipients to promote the extension of the conjugation machinery from the donor to the recipient cell and facilitate membrane fusion. The CHAP domains presented some conserved sequences from the core of other Gram-positive CHAP amidase domains, such as PcsB, an essential putative peptidoglycan hydrolase from *Streptococcus pneumoniae* (Rigden *et al*., 2003; Sham *et al*., 2011), suggesting these are functional and act as the catalytic domains of both TraAd and TrsAd. Based on our AlphaFold2 structure predictions, the catalytic triad of domain 2 from both TraAd and TrsAd (the CHAP domains) is formed by Cys/His/Asp-residues. Other previously characterized CHAP domains present similar catalytic triads but with Glu-as the last residue, like the proteolytic triad formed by Cys/His/Glu-in the CHAP domain of *Staphylococcus saprophyticus* (Rossi *et al*., 2009) or the Cys/His/Glu-catalytic triad present in the CHAP domain of the endolysin LysK from *Staphylococcus aureus* bacteriophage K (Sanz-Gaitero *et al*., 2014).

Our results indicate that the N-terminal peptidoglycan hydrolase domains act as the substrate-binding domains of both proteins, although no significant similarities to other peptidoglycan-binding proteins were found. However, additional experimental data will be required to propose a more detailed mechanism of action, and future studies will focus on the elucidation of the function and mode of action of TraAd and TrsAd. Introducing mutations in the proposed catalytic sites of each peptidoglycan-active domain will facilitate an evaluation of the effect of such mutations on the *in trans* complementation and cell clumping. Additionally, it will provide insights into the domains that are essential to establish cell binding, which are currently unknown. Further assays should also determine if TraAd and TrsAd possess the hereby proposed cell wall-degrading activity, while transmission electron microscopy analysis may provide key insights into the exact effect that these proteins have on the cell walls and membranes of donor and recipient cells.

The work described herein provides a promising starting point for future studies on lactococcal plasmid-encoded adhesion proteins. In turn, this could enhance efforts to boost conjugation frequencies and facilitate the generation of more robust starter cultures in the dairy industry using means of natural transformation.

## CRediT authorship contribution statement

**Guillermo Ortiz Charneco:** Conceptualization, Methodology, Validation, Formal analysis, Investigation, Writing – original draft, Writing – review & editing, Visualization. **Philip Kelleher:** Conceptualization, Software. **Andrius Buivydas:** Conceptualization. **Paul P. de Waal:** Conceptualization, Writing – review & editing, Funding acquisition. **Irma M.H. van Rijswijck:** Conceptualization, Writing – review & editing, Funding acquisition. **Noël N.M.E. van Peij:** Conceptualization, Writing – review & editing, Funding acquisition. **Christian Cambillau:** Software, Visualization. **Jennifer Mahony:** Conceptualization, Validation, Writing – review & editing, Supervision, Project administration, Funding acquisition. **Douwe Van Sinderen:** Conceptualization, Validation, Writing – review & editing, Supervision, Project administration, Funding acquisition.

## Declaration of competing interest

PdeW, IvR & NvP are employed by the company dsm-firmenich. The remaining authors declare that the research was conducted in the absence of any commercial or financial relationships that could be construed as a potential conflict of interest.

## Data Availability

Data will be made available on request.

## Appendix Supplementary materials

**Supplementary Figure S1.** Comparative gene map of the conjugation gene clusters of pNP40 and pUC11B. The figure details the genetic organization of both conjugation gene clusters and their shared amino acid identity (indicated by shaded areas). The predicted conjugation-related functions (Ortiz Charneco *et al*., 2023b) are indicated by coloured arrows. Image, application 1

**Supplementary Figure S2.** Excel plot of the predicted Local Distance Difference Test (pLDDT) displaying domains 1 and 2 from TraAd **(A)** and TrsAd **(B)**. Image, application 2

Image, application 3

Image, application 4

Image, application 5
