# Supplementary material for: Discovering genetic determinants for cell-to-cell adhesion in two prevalent conjugative lactococcal plasmids
Source: Curr Res Microb Sci. 2024 Apr 23;6:100239. doi: 10.1016/j.crmicr.2024.100239 (PMC11067333; doi:10.1016/j.crmicr.2024.100239)
Supplement: Supplementary file 3 [file mmc3.docx]

**Supplementary Table S1.** Lactococcal strains used in this study.

| **Strain** | **Plasmids present in the strain** | **Relevant properties** |
| --- | --- | --- |
| **L. cremoris MG1614** | - | Main recipient strain, plasmid-free and streptomycin resistant |
| **L. cremoris NZ9000** | - | Derivative of the parental strain *L. cremoris* MG1363 |
| **L. cremoris NZ9000** | pNP40 | Strain harbouring the conjugative plasmid pNP40 (Nis^r^) |
| **L. cremoris NZ9000** | pUC11B | Strain harbouring the conjugative plasmid pUC11B (Tc^r^) |
| **L. cremoris NZ9000** | pPEPi | Strain harbouring the inducible low copy vector pPEPi (Erm^r^) |
| **L. cremoris NZ9000** | pPTPi | Strain harbouring the inducible low copy vector pPTPi (Tc^r^) |
| **L. cremoris NZ9000** | pPEPi::*traAd* | Strain overexpressing *traAd* in pPEPi |
| **L. cremoris NZ9000** | pPEPi::*trsAd* | Strain overexpressing *traAd* in pPEPi |
| **L. cremoris NZ9000** | *traAd*::Ter | Strain harbouring the *traAd* mutant derivative of plasmid pNP40 |
| **L. cremoris NZ9000** | *trsAd*::Ter | Strain harbouring the *trsAd* mutant derivative of plasmid pUC11B |
| **L. cremoris NZ9000** | pPTPi, pGFP8048E | Strain harbouring low copy vector pPTPi (Tc^r^) and pGFP8048 (Erm^r^) expressing GFP under the control of the nisin inducible promoter from pNZ8048E |
| **L. cremoris NZ9000** | pPTPi, pMC8048E | Strain harbouring low copy vector pPTPi (Tc^r^) and pMC8048 (Erm^r^) expressing mCherry under the control of the nisin inducible promoter from pNZ8048E |
| **L. cremoris NZ9000** | pPTPi::*traAd*, pGFP8048E | Strain harbouring low copy vector pPTPi overexpressing *traAd* from pNP40 (Tc^r^) and pGFP8048 (Erm^r^) expressing GFP under the control of the nisin inducible promoter from pNZ8048E |
| **L. cremoris NZ9000** | pPTPi::*trsAd*, pMC8048E | Strain harbouring low copy vector pPTPi overexpressing *trsAd* from pUC11B (Tc^r^) and pMC8048 (Erm^r^) expressing mCherry under the control of the nisin inducible promoter from pNZ8048E |
