# Supplementary material for: Discovering genetic determinants for cell-to-cell adhesion in two prevalent conjugative lactococcal plasmids
Source: Curr Res Microb Sci. 2024 Apr 23;6:100239. doi: 10.1016/j.crmicr.2024.100239 (PMC11067333; doi:10.1016/j.crmicr.2024.100239)
Supplement: Supplementary file 4 [file mmc4.docx]

**Supplementary Table S2.** Plasmids and plasmid constructs employed in this study.

| **Plasmid** | **Characteristics** | **Reference** |
| --- | --- | --- |
| **pNZ44E** | Erythromycin-resistant, *E.coli*-*L. lactis* shuttle vector pNZ44, with a P44 constitutive promoter | Draper *et al*., 2009 |
| **pNZ8048E** | Erythromycin-resistant version of the high-copy-number *E. coli*-*L. lactis* overexpression vector, P*nisA;* Erm^r^ | Ortiz Charneco *et al*., 2024 |
| **pGFP8048E** | Erm^r^ derivative of plasmid pNZ8048, expressing GFP under the control of a nisin-inducible promoter, P*nisA* | This study |
| **pMC8048E** | Erm^r^ derivative of plasmid pNZ8048, expressing mCherry under the control of a nisin-inducible promoter, P*nisA* | This study |
| **pPTPi** | Low-copy-number *E. coli-L. lactis* vector, Tc^r^, P*nisA*, pPTP derivative | O’Driscoll *et al*., 2004 |
| **pPEPi** | Erythromycin derivative of the low-copy-number *E.coli*-*L. lactis* shuttle vector, pPTPi | Ortiz Charneco *et al*., 2023 |
| **pPEPi::traAd-trsAd** | pPEPi constructs containing a copy of the possible surface adhesins from the pNP40 (*traAd*) or the pUC11B (*traAd*) conjugation gene clusters | This study |
| **pPTPi::traAd-trsAd** | pPTPi constructs containing a copy of the possible surface adhesins from the pNP40 (*traAd*) or the pUC11B (*trsAd*) conjugation gene clusters | This study |
