# Supplementary material for: Discovering genetic determinants for cell-to-cell adhesion in two prevalent conjugative lactococcal plasmids
Source: Curr Res Microb Sci. 2024 Apr 23;6:100239. doi: 10.1016/j.crmicr.2024.100239 (PMC11067333; doi:10.1016/j.crmicr.2024.100239)
Supplement: Supplementary file 5 [file mmc5.docx]

**Supplementary Table S3.** Oligonucleotides used in this study.

| Oligonucleotide name | Oligonucleotide sequence (5’ → 3’) |
| --- | --- |

| Surface adhesin cloning primers^ᴪ^ |  |
| --- | --- |
| TraAd-Fw | AGCAGCGGATCCAGGAGGCACTCACCATGGCAGGAAAGTATATTTTTAA |
| TraAd-Rv | AGCAGCGAATTCATTTATTTTCCATGCTGTGCCT |
| TrsAd-Fw | AGCAGCGGATCCAGGAGGCACTCACCATGTTTGTAACCAAAGCAAAAATA |
| TrsAd-Rv | AGCAGCGAATTCTTTTCTTTTCCATAGCTTATTTTC |
|  |  |
| GFP cloning primers^ᴪ^ |  |
| GFP-Fw | AGCAGCCTGCAGAGGAGGCACTCACCATGGGAGTTAGCAAAGGTGAA |
| GFP-Rv | AGCAGCGAGCTCTTATTTGTACAGTTCATCCATGCC |
|  |  |
| mCherry cloning primers^ᴪ^ |  |
| mCherry-Fw | AGCAGCGGTACCAGGAGGCACTCACCATGAGCAAGGGCGAGGAGGAT |
| mCherry-Rv | AGCAGCGAGCTCTTACTTGTACAGCTCGTCCATGC |

ᴪ Introduced restriction enzyme sites are single underlined, whereas, Shine-Dalgarno sequences from pNZ8048 are double underlined.
